# Probiotic model for studying rhizosphere interactions of root exudates and the functional microbiome

**DOI:** 10.1093/ismejo/wrae223

**Published:** 2024-11-04

**Authors:** Zhiqiang Pang, Peng Xu

**Affiliations:** CAS Key Laboratory of Tropical Plant Resources and Sustainable Use, Xishuangbanna Tropical Botanical Garden, Chinese Academy of Sciences, Mengla, Yunnan 666303, P. R. China; College of Life Sciences, University of Chinese Academy of Sciences, Beijing 101408, P. R. China; CAS Key Laboratory of Tropical Plant Resources and Sustainable Use, Xishuangbanna Tropical Botanical Garden, Chinese Academy of Sciences, Mengla, Yunnan 666303, P. R. China; College of Life Sciences, University of Chinese Academy of Sciences, Beijing 101408, P. R. China

**Keywords:** plant–microbes interaction, coevolution, root mucilage, plant microbiome, microbial homeostasis

## Abstract

Root exudates are important mediators of plant–microbiome interactions. Recent pioneering studies on various aerial root plants, including cereals, have shown that carbohydrate-rich mucilage can enrich diazotrophs and increase host nitrogen utilization and growth. Moreover, non-diazotrophic “gatekeeper” microorganisms in mucilage help defend against pathogenic and environmental microbes. These findings highlight the active role of root exudates in mediating plant–microbiome interactions to maintain microbial homeostasis in the rhizosphere. However, little is known about the specific mechanisms by which root exudates modulate the functional microbiome and homeostasis in rhizosphere microhabitats. Here, we propose the typical and stable biointeractions of four plant–specific aerial root mucilage–probiotic systems as a model for understanding root exudate–functional microbiome interaction. We anticipate that this model can provide fundamental biological insights into rhizosphere interactions.

## Graphical Abstract

**Graphical Abstract:**
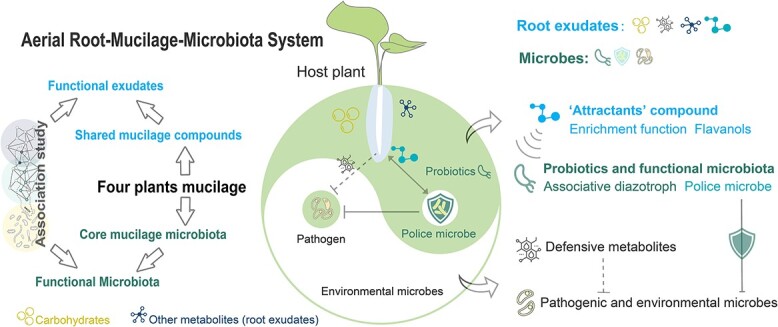

## Introduction

Roots secrete many metabolites/exudates into the “rhizosphere” (see Glossary; herein, the rhizosphere includes the aerial/underground areas around the root system), including primary and secondary metabolites, such as sugars and other photosynthates, acids, terpenes, alkaloids, and flavonoids [1, 2]. These root metabolites/exudates are released into the environment to participate in a series of rhizosphere processes [3–5], such as shaping plant–microbiome interactions, plant growth and adaptation. Studies have shown that root exudates shape the microbiome which in turn modulates host growth and health [2, 6]. The root exudate components, especially some benzoxazinoids, flavones, coumarins, terpenes, and volatile organic compounds (VOCs), modulate the rhizosphere microbiome to improve plant growth in adverse environments [2, 7, 8].

Previous studies have provided insights into specialized root exudates for their ability to defend against environmental pathogens and maintain microbial “homeostasis” (see Glossary) in host habitats and plant health [9, 10]. Microbial homeostasis may be critical for plant health and stress tolerance, suggesting that plants have evolved mechanisms to prevent dysbiosis and support a health–promoting microbiota under stress [11]. However, many questions remain to be explored regarding how host exudates modulate microbiome assembly, shape the “functional microbiome” (see Glossary), and maintain microbial homeostasis.

Advances box

- Root exudates mediate interactions with environmental microbiota, shaping microbial functions, and promoting host growth.
- Various aerial root plants secrete carbohydrate-rich mucilage that provide exudates to feed the functional microbiome, promoting biological nitrogen fixation (BNF) and enhancing plant growth.
- How root exudates shape the functional microbiome and maintain microbial homeostasis remain poorly understood.
- To investigate the mechanisms by which the plant functional microbiome is modulated and microbial homeostasis is maintained, we propose a novel aerial root mucilage-functional microbiota system of four plants, including *Poaceae* multi–cereal plants, as a model for studying the interaction mechanism between root exudates and the functional microbiome.

## Aerial root mucilage is a typical root exudate

**Figure 1:**
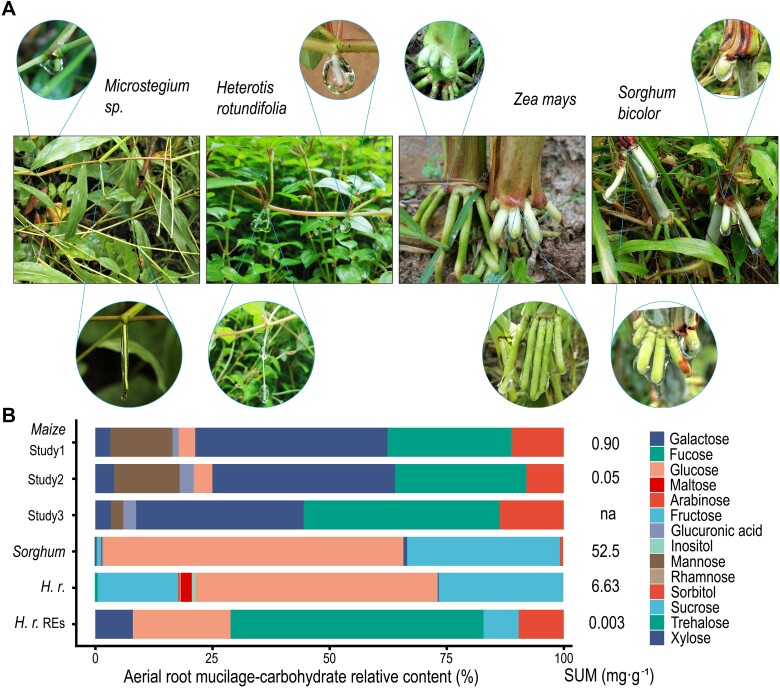
Four aerial root mucilage plants and mucilage carbohydrate content. (A) Three *Poaceae* (*Zea mays*, *Sorghum bicolor*, and *Microstegium Nees* are monocotyledons) and one *Melastomataceae* vine plant (Pink Lady *Heterotis rotundifolia* is a dicotyledon), whose aerial roots secrete large amounts of mucilage in Xishuangbanna (Southwest China). (B) Mucilage carbohydrate content of different plants. For maize studies 1–3 and the sorghum and *H.r.* data, refer to Table S1.

**Figure 2:**
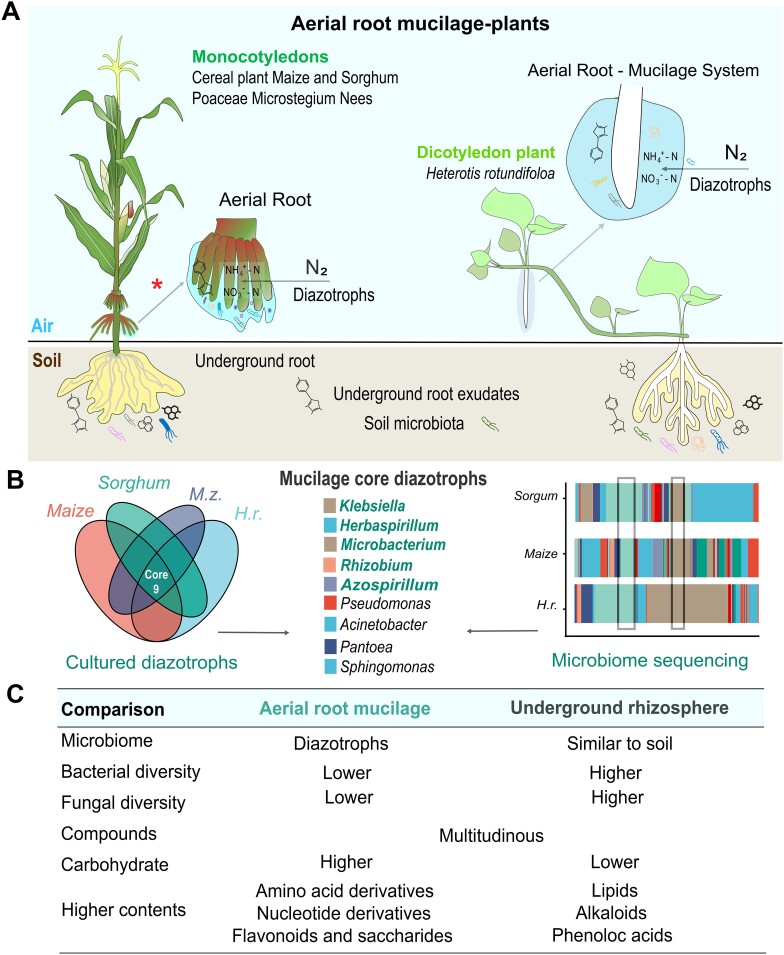
Comparison of the metabolites and microbiome of aerial root mucilage and underground rhizosphere*.* (A) Aerial root mucilage diazotroph system of monocotyledon (*Poaceae* crops) and dicotyledon (*Melastomataceae*) plants. These plants could secrete mucilage from aerial roots to enrich diazotrophs for nitrogen fixation and plant growth (up to 54.8%–82% of BNF). (B) Core diazotrophic taxon of different aerial root-mucilage-plants. These diazotrophs are derived from published data of *Poaceae* crops, and we have isolated them from the mucilage of four plants species. (C) Analysis of different compounds in aerial root mucilage and underground root exudates. Note: The *Poaceae* diagram was referenced from Guo [19] and modified again.

Aboveground aerial roots carry out respiration, support photosynthesis, and other root biological functions like underground root [12, 13]. Aerial root tips exude mucilage to protect root tip cells and help plants enter the soil or climb vertical surfaces of plant/other supporters. We identified four monocotyledonous and dicotyledonous plants (*Poaceae* and Pink Lady plants) whose aerial roots secrete a large amount of mucilage exudates in Xishuangbanna, Southwest China (Fig. 1A). This aerial root mucilage is produced from plant stems 13–140 cm above the ground and varies from 0.1 cm to >20 cm in length. Recent studies have shown that this mucilage is rich in primary and secondary metabolites (Table S1), containing 78.4% polysaccharides, 7.3% proteins, 5.6% minerals, and 3.1% lipids [14]. In particular, the carbohydrates fucose, galactose, arabinose, glucose, and special heterogeneous polysaccharides in *Poaceae* maize and sorghum are prevalent (Fig. 1B) [15–17]. Our previous study on dicotyledon plants revealed that root mucilage is also enriched in flavonoids and saccharides, significantly differing from underground root exudates, which are enriched in higher contents of lipids, alkaloids, and phenolic acids (Figs 2C, S1 and Table S1) [18–19]. However, the specific compound composition of aerial root mucilage in this special niche has not been thoroughly analyzed and studied.

Carbohydrate-rich mucilage provides abundant carbon and nutrients, moist environments, and protective barriers for microorganisms and biological interactions [20]. In addition, the high carbon content and low oxygen availability in the mucilage suggest that this system may involve in microbial interaction and BNF [5]. Pioneering study has found that the aerial root–mucilage system can fix atmospheric nitrogen and promote the growth of multiple plants (Fig. 2A) [15, 18, 21]. Thus, carbohydrate-rich mucilage-aerial root, which differs from underground rhizosphere may be related to new biological functions (Fig. 2C). In this work, we discuss these new biological functions and the maintenance mechanisms of aerial root mucilage and provide new insights into the interactions between root exudates and their functional microbiome.

## Diazotrophs are present in the aerial root mucilage of distinct host plant species

The aerial root mucilage of different plants is enriched in carbohydrates and various nutrients, which serve as natural media for enriching microbial communities. Previously, a pioneering and systemic study of Mexican maize revealed that aerial root mucilage was enriched in the bacteria taxa including *Raoultella*, *Klebsiella*, and *Azospirillum* (Fig. 2A and B) [15, 21–24]. Subsequent amplicon sequencing and microbial culture analyses revealed similar microbial community structure across different habitats, highlighting potential diazotrophs, such as the high-abundance genera *Klebsiella*, *Herbaspirillum*, *Microbacterium*, and *Azospirillum* (Figs 2A and B and S2) [18, 22, 23]. These diazotrophs are widely used as model systems for studying associative nitrogen fixation and are well utilized in agricultural production [25]. Furthermore, studies from around the globe in different plants have shown that the aerial root–mucilage system can directly fix atmospheric nitrogen for host utilization and support growth based on microbial functional annotation, BNF assays and molecular evidence (Fig. 2A) [15, 18, 21–23, 26].

The enrichment of potential diazotrophs in aerial root mucilage suggests that the mucilage–microbiome system may be crucial for nitrogen fixation in these plants. Similar diazotrophs have been isolated from the aerial root mucilage of different plants worldwide, suggesting that carbohydrate-rich and low-oxygen mucilage is an ideal niche for diazotrophs (Fig. 2B) [18, 21–23, 27, 28]. However, the mechanisms by which hosts select these functional diazotrophs and maintain their function in the rhizosphere mucilage microenvironment remain unclear. There is a need for specialized experimental models to advance our understanding of how plant metabolites mediate host–functional microbiome interactions.

## Microbial homeostasis in aerial root mucilage microhabitats

How host plants modulate their microbial community and maintain probiotic and pathogen homeostasis to maximize plant fitness remains a key topic. In the past few decades, studies have shown that host factors and microbial interactions maintain microbial homeostasis and host health [29–34]. However, little is known about the modulatory factors in maintaining microbial homeostasis. Similarly, how the mucilage microenvironment is enriched with carbohydrates/carbon nutrients while avoiding outbreaks of environmental/pathogenic microorganisms, and maintaining microbial function and homeostasis is worth considering. We previously found that the microbial diversity of aerial root mucilage is lower than in rhizosphere and bulk soil, with a specialized microbial community in mucilage exhibiting higher abundances of diazotrophic taxa rather than pathogenic and environmental microbes [18] (Fig. 2C). Additionally, the microbial community structure in maize remains consistent across different developmental stages and planting sites [27], suggesting that microbial stability and maintenance of microbial homeostasis maintenance phenomena exist within the mucilage.

Our previous research demonstrated that microbial homeostasis in the mucilage microenvironment is driven by internal members: a gatekeeper (police) fungus can specifically defend against both pathogenic and environmental microbes rather than sympatric diazotrophs [18]. This establishes a beneficial partnership within the aerial root mucilage microhabitat. The “gatekeeper microbe” (see Glossary) and specific mucilage compound (although not at physiologically relevant concentration) have antibiotic functions against specific microbes [18]. This finding deepens our understanding of the role of microbial homeostasis in the protection of the functional microbes and host plant adaptation. Therefore, it is necessary to focus on the modulatory mechanisms of the functional microbiome and homeostasis in rhizosphere microhabitats. Mucilage as a special rhizosphere microenvironment provides a new perspective for studying stable biointeraction and microbial homeostasis.

## New model of root exudate–rhizosphere functional microbiome interactions

**Figure 3:**
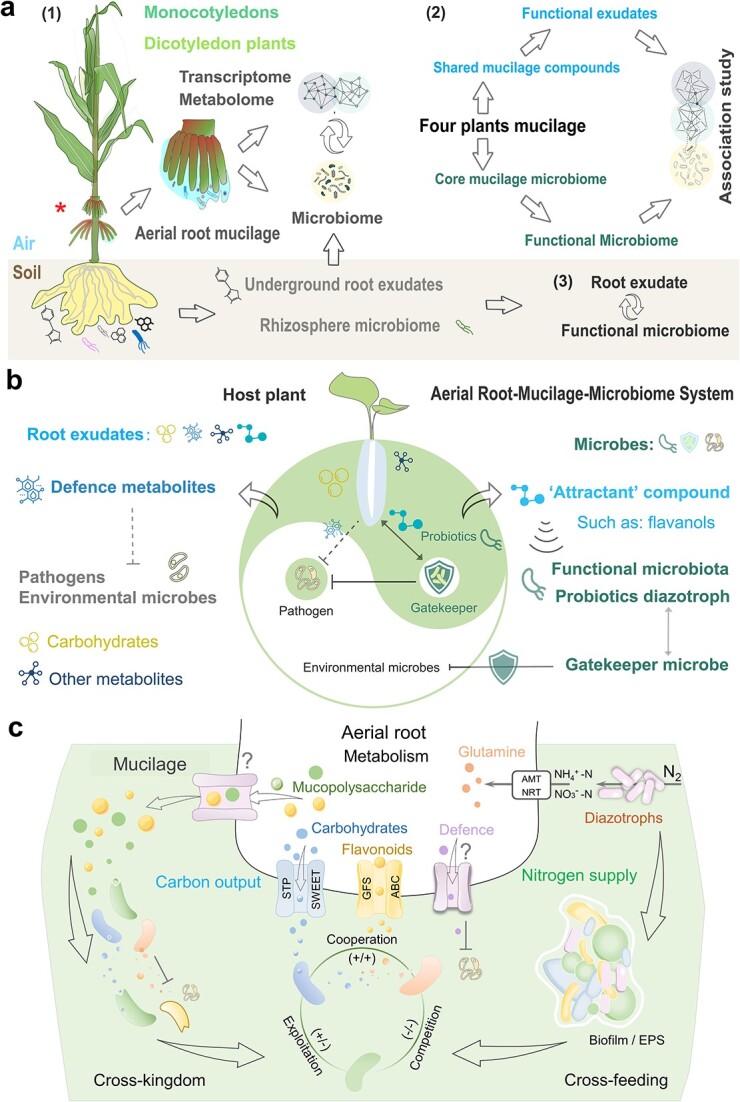
Proposed model of root exudate–functional microbiome interactions and research approach. (A) Linking the functional components of exudates and the functional microbiome of mucilage. Analyzing the metabolites compounds and microbiome components of aerial root mucilage in four different plant species to identify common components, and analyzing the differences components of metabolites and microbiome between aerial root mucilage and underground root exudates of the same species to identify the unique components of aerial root mucilage. Finally, validating the interactions between common and differential components of metabolites and microbiome. (B) The exudate (mucilage) microenvironment and gatekeeper microbes play crucial roles in maintaining homeostasis, which helps the functional microbiome enrichment and defense against pathogen invasion. (C) Microbial interactions mediated by mucilage compounds. Mucilage, as a typical carbohydrate, can be used by different microbiome. Mucopolysaccharides, carbohydrates, flavonoids, and defense (antimicrobial substances) can be secreted into the environment by different transporters in the host root system to cause microbial utilization and mediate microbial interactions. At the same time, the host uses nitrogen transporters to assimilate nitrogen (ammonium and nitrate) which fixed by mucilage diazotrophs to further synthesize amino acids. In addition, microbial metabolites may be used or defend other microbial members, leading to different microbial interactions.

The interaction between host specialized metabolite and the microbiome is a key factor in modulating rhizosphere microbiome assembly. Understanding the mechanism by which root exudates shape and modulate the plant-associated microbiomes is critical for ensuring plant growth and health. Based on the typical and stable interactions of aerial root mucilage-functional microbiome in multiple hosts and areas [15, 18, 21], we propose the aerial root mucilage-probiotic system as a new model for studying root exudate–functional microbiome interactions. In this model, three *Poaceae* monocotyledons and one *Melastomataceae* dicotyledon vine plant (Pink Lady) were used to identify the similarities and differences in metabolites and microbiome in the aerial root mucilage and underground rhizosphere (Fig. 3A). The functional microbiome contains probiotics and gatekeeper microbes, which either serve as diazotrophs or help maintain microbial homeostasis in the aerial root mucilage microhabitat.

**Figure 4:**
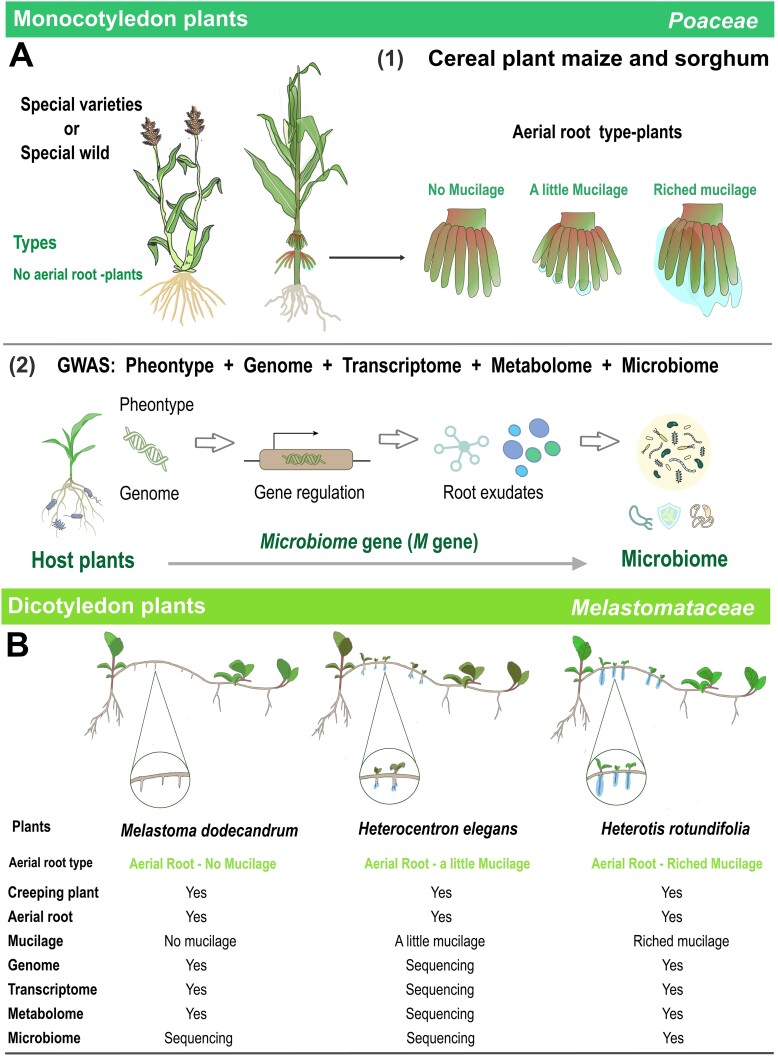
Occurrence and evolution of aerial root-mucilage-microbiome traits in plants. (A) Multiomics approaches, including genome, transcriptome, metabolome, and microbiome analyses, were used to investigate the formation of aerial root, the compounds and function of aerial root mucilage in monocotyledon (*Poaceae*) crops. The microbiome can be regarded as the second genome of the host plants and special traits. The potential host genetic factors modulating the functional microbiome can be identified by GWAS. The *Poaceae* diagram was referenced from Guo [19] and modified. (B) Multiomics sequencing data of three aerial root mucilage dicotyledon (*Melastomataceae*) plants. These small plants with aerial roots can be further used for nitrogen isotope labeling experiments.

Our scientific hypotheses and proposed solutions for this model involve four primary criteria: (i) Identify commonalities in mucilage metabolites and microbiome to establish connections between root exudates and the functional microbiome: analysis of common metabolites and core microbiome in different aerial root mucilage-plants. The differences in exudates and microbiome between mucilage and underground rhizosphere microenvironment can also provide clues for finding functional components (Fig. 3A—[1]). (ii) Validate interactions between root exudates and the functional microbiome by linking metabolites to microbiome functions, demonstrating how exudates shape probiotics, and how the functional microbiome metabolizes mucilage components (Fig. 3A—[2]). (iii) Identify the functional composition of the mucilage microenvironment, including key metabolites and microbiome that modulate functional microbiome and microbial homeostasis (Fig. 3B). (iv) Analyse the evolution of aerial root mucilage formation and microbial interactions in multiple plants from an eco-evolutionary perspective (Fig. 4). Multi-omics methods (transcriptome, metabolome, and microbiome) combined with experimental verification can be used to investigate the interactions between host–mucilage metabolites and microbiome, elucidating modulatory mechanisms and microbial homeostasis in the rhizosphere niche. This model provides a new perspective on the microbial functions and maintenance mechanisms of specific exudate mucilage and rhizosphere microenvironments, and helps identify host modulatory factors and microbial interactions shaping the microbial community, function and homeostasis (Fig. 3).

## Linking functional exudate components and the functional microbiome

Identifying the key metabolites of root exudates and their modulatory mechanism on the functional microbiome is important for plant health and growth. Our model provides a new perspective for identifying the key mucilage compounds that modulate the functional microbiome and/or defend against pathogenic and environmental microbes. These special exudate components are defined as the “functional metabolites” (see Glossary), similar to flavonoids, which are essential plant signaling molecules that modulate legume symbiosis with rhizobia, and shape the interaction between the root-associated microbiome in nonleguminous plants [35–37]. A pioneering study showed that flavonoids secreted by maize roots recruit specific microbes, such as *Oxalobacteraceae*, to promote root development and plant growth under low-nitrogen conditions [7]. This suggested that flavonoids may be important components of nonleguminous root exudates by recruiting associated diazotrophs (plant growth-promoting bacteria, PGPR, or diazotrophs). Moreover, rice root exudates, such as apigenin and flavonoids, induce nitrogen-fixing bacteria to produce biofilms and enhance nitrogen fixation and promote host growth [38]. Transcriptome data from mucilage-producing aerial roots further revealed elevated expression of genes involved in flavonoid synthesis and carbohydrate metabolism than non-mucilage aerial roots (unpublished). Therefore, to clarify the mechanisms of plant–functional microbiome interactions, driven by host produced-metabolites, we propose using the aerial root mucilage probiotics of four plant species as a novel model to study the crucial interactions between functional metabolites and the microbiome, and to determine how probiotics are selected within this system (Fig. 3A).

In this exudate–functional microbiome interaction model, one type of functional metabolite defined as an “attractant” is an exudate compound or nutrient that enriches the functional microbiome (Fig. 3B). These specialized metabolites can increase the abundance of selected functional microbiome members in the rhizosphere. Our previous study provided a list of candidate “modulator” functional compounds in mucilage microenvironments, including flavanols (e.g. epigallocatechin-3-gallate, epicatechin, naringenin-7-O-neohesperidoside, and naringin), tannins (e.g. gallic acid), amino acids and derivatives (e.g. L-tyrosine, L-proline, L-homoserine, L-threonine, L-tryptophan, L-valine, trans-4-hydroxy-L-proline, O-acetylserine), and carbohydrates [18]. Moreover, pioneering opinions listed candidate mucopolysaccharides involved in these interactions and explored their potential applications in sustainable agriculture from an engineering perspective [39]. These functional compounds and mucilage mucopolysaccharides may replicate optimal rhizosphere environments to maintain specific microbiota, such as the functional diazotrophs. They provide essential carbon and nitrogen sources, supporting the functional microbiome metabolism, and facilitate basic colonization mechanisms within the root mucilage microenvironment [5]. To assess whether these compounds can stimulate microbial proliferation and biofilm formation, thereby improving stable colonization, further studies are needed (Fig. 3C). These compounds can be utilized by the functional microbiome through potential cross-feeding processes. Experiments using in vivo/*vitro* systems and synthetic microbial communities (SynComs) can help verify these interactions (Fig. 3C). Pioneering insights have provided information related to the molecular mechanisms of plant–functional microbiome (such diazotrophs) interactions [39], suggesting the importance of exploiting the power of plant metabolites in the design of mucilage functional diazotrophs associations.

Mucilage, rich in carbohydrates, creates a natural medium and ideal ecological niche for the functional microbiome, but it may also be susceptible to pathogenic and other environmental microbes. One important scientific question is how the mucilage microenvironment maintains microbial homeostasis, promoting functional microbe colonization, while preventing pathogen invasion (Fig. 3B). Studies have shown that specific root exudates modulate microbial communities and prevent pathogenic invasion [7, 33, 40, 41]. This model provides new clues for identifying specific functional metabolites that act as attractants for the functional microbiome (such as prebiotics and signaling compounds), while also functioning as defense compounds with antibacterial activities against pathogens. For instance, in legume-rhizobia symbiosis, flavonoids exudates not only recruit rhizobia but also inhibit other microbes in the rhizosphere [42]. Our model, along with previous studies, have provided a range of candidate defense compounds, such as naringenin-7-O-neohesperidoside (Naringin), stearic acid, α-linolenic acid, γ-linolenic acid, quinic acid, ursolic acid, choline, spermine, sallic acid, flavonoids, and phthalic anhydride [18]. In summary, mucilage may contain functional attractor compounds and antimicrobials defense metabolites. Plants synthesize these functional compounds and secrete into the rhizosphere by root transporters (Fig. 3C). At the same time, the host uses other element transporters to assimilate and absorb nutrients provided by rhizosphere functional microbiome. The proposed study model will contribute to expand and define these candidates.

Mucilage metabolites may first originate from host plants and then be metabolized to other metabolites by various microorganisms (Fig. 3C), highlighting the importance of considering dynamic metabolites composition and cross-feeding within the mucilage microbiome. Mucilage shares physical and chemical properties with extracellular polymeric substances (EPS), providing a protected habitat for nutrient mobilization [20]. Its composition and function may also resemble mammalian gut mucus, which contains mucins, milk oligosaccharides, and glycoconjugates. Moreover, mucilage diazotrophs such as *Azospirillum* may decompose complex polysaccharides into carbon sources for energy by glycosyl hydrolases [5]. Our model provides insights into how the microbial community can be modulated by carbohydrates, metabolites, and interactions, such as cross-kingdom and cross-feeding processes (Fig. 3C). Future studies could exploit these microbial interactions, particularly those between mucilage-breaking down microbes and other functional microbiome.

## Microbial interactions shape microbiome homeostasis

An alternative explanation for how the microenvironment maintains homeostasis is through selective suppression, where certain microbes suppress undesirable environmental microbes while allowing sympatric functional microbes. However, studies on the microbial interactions driving homeostasis in the rhizosphere remain limited, and mechanisms governing this balance are not well understood. An important topic is microbial homeostasis and dysbiosis, although the causal relationship between ecological improvement and host health status remains poorly understood [11, 43]. In the aerial root mucilage system, considering only how the mucilage microenvironment maintains microbial homeostasis simplifies the experimental design, as the impact on host health can be set aside; mucilage itself may serve as a physical barrier.

Therefore, this exudate model provides a microecological perspective on sympatric microbial interactions that drive homeostasis between functional and pathogenic microbiomes (Fig. 3B). The carbohydrate-rich composition of the root mucilage microhabitat provides an ideal study model and a natural medium to explore this topic in depth. Identification of the key gatekeeper microbes that protect against pathogens and interact with sympatric diazotrophs is highly valuable for understanding the microbial homeostasis of the rhizosphere microenvironment. Moreover, colonization resistance, a collective property of microbial communities, can emerge through the synergistic action of multiple strains [44]. Making it necessary to understand whether microbial interactions in the mucilage niche involve cooperation (e.g. enhanced mucilage utilization or functional cooperation), or antagonism (e.g. pathogen defense) and other cross-kingdom interactions [44–46]. The beta diversity of the microbial composition was determined using the Anna Karenina Principle to identify the functional status, dysbiosis and the core components of microbial homeostasis in the mucilage microbiome [43]. Therefore, probiotics, gatekeeper microbes and environmental microbes isolated from mucilage can be used to establish a SynCom for studying microbial homeostasis and sympatric biointeractions (Fig. 3B and C).

The synergistic effects of host-specific compounds (root exudates) and the functional microbiome can be further explored to clarify the potential contributions of host metabolite and microbiome interactions to maintain homeostasis in the rhizosphere microenvironment (Fig. 3C). For example, root mucilage, which provides a biofilm-like environment similar to EPS, facilitate bacterial communication and function in the rhizosphere. This highlights the potential of plant mucilage to act as a biofilm [20]. As mentioned above, the interactions between mucilage metabolites and breakdown microbes, the functional microbiome, and other environmental microbes in this model deserve further attention (Fig. 3C). These relationships may be common in plant–microbiome interactions, with fundamental practical implications.

## Evolution of aerial root mucilage and coevolution of the plant microbiome

The mechanisms of host control of the microbiome are diverse across different plant and animal species [47, 48]. Evolutionary biologists have focused on the occurrence and evolution of plant organs to obtain more information about the coevolution of plant–microbe symbionts adapting to the environment. Similarly, botanists and microbiologists have attempted to elucidate the evolution of rhizobial symbioses, arbuscular mycorrhizal symbionts, and microbial adaptations to plants [48–52]. However, it is not clear how the plant-associated microbiome was constructed during plant evolution and microevolution (including plant–microbiome microevolution of artificial domestication). Compared with the gut microbiome research, few studies have identified the dynamic processes and cascade effects of the interactions among host plant genes (e.g. “the *microbiome* genes”, or “*M* genes”; see Glossary), root exudates, and the functional microbiome in plants, and the underling mechanisms are still unclear. Aerial root mucilage, as a specialized root exudate with similarities to intestinal mucilage, offers a potential avenue for future exploration.

Little is known about the effects of plant phylogeny and trait evolution on the dynamics of host–microbiome coevolution. And the host genetic regulators and mechanisms used by plants to control aerial root mucilage (or root exudates) and the abundance of beneficial microbes are largely unknown. Previous studies found that one key *ZmSBT3* gene and a UDP-glycosyltransferase gene are negative regulators of mucilage secretion and nitrogen fixation in maize and sorghum roots [17, 26]; and one gene has also been reported to be involved in mucilage secretion by the seed coat in *Arabidopsis thaliana* [53]. In these maize and sorghum studies, aerial root and mucilage production were used as a plant trait factor to identify potential regulatory factors via genome–wide association study (GWAS) [17, 26]. Unfortunately, this aerial root mucilage trait may gradually lost during maize domestication [15, 17, 26]. Only a limited number of modern maize inbred lines (such as HN5-724) retain this trait [26]. Thus, it is necessary to investigate the genomic–phenotypes effect of many modern and ancient varieties and wild species and to identify the potential processes regulating other interaction traits (e.g. microbiome). In addition, the identification of host genetic elements associated with the plant–microbe interaction is an ongoing topic of plant–microbe interaction research. Multiple studies have used different plant and genome-wide association analyses to identify potential key host regulators [54–57]. Therefore, we further proposed a potential evolutionary biology model for the occurrence and evolution of this trait of the aerial root mucilage microbiome in monocotyledon (*Poaceae* crops) and dicotyledon (*Melastomataceae*) plants (Fig. 4).

In this plant–functional microbiome coevolution model, we identified those plants whose aerial root development and mucilage production traits were selected as candidates. For example, sorghum and maize with different aerial root/mucilage traits and their wild species, farm varieties and cultivars in *Poaceae* crops (Fig. 4A—[1]), and three related *Melastomataceae* plants with similar traits were selected as study materials (Fig. 4B). We proposed the aerial root mucilage-microbiome-system as a special host developmental trait and linked the host genome, gene expression, metabolite synthesis and transport, and other multitrait analyses to identify the potential cascade effect of the host genotype–functional gene–root exudates regulating the plant-associated microbiome. In this model, four related types of plants, with or without aerial roots, presented different aerial root mucilage traits and exhibited changes in the same habitat (Fig. 4A—[2]).

Manipulating the microbiome to improve host growth and health is challenging. The model also included identification of the “*microbiome* genes” (“*M* genes”, see Glossary). The large number of gramineous genomes, metabolome, and genetic transformation system will provide insights into *M* genes and the identification of trait occurrence in aerial root–mucilage and mechanisms involved in host–microbiome interactions. In addition, the short generation time also means that microbiome can rapidly evolve and undergo genetic changes within its population during the lifetime of the host, and the properties of the host microbiome selected by the mucilage environment, such as the ability to metabolize carbohydrates mucilage and that favor the host, warrant further consideration [47].

These *Melastomataceae* small plants with aerial roots can be further used for nitrogen isotope labeling experiments and multiomics assays to determine the contribution of mucilage–functional microbiome to plant nitrogen uptake and potential molecular and physiological evidence for the occurrence of aerial root mucilage traits [18]. For example, genome, microbiome, (meta) transcriptome, and metabolome samples from three related dicotyledonous plant species were selected for sequencing and analysis (Table S2). This model will help us understand the occurrence and evolution of aerial root mucilage traits in plants and relate them to clues about the host’s regulatory effects on the microbiome. This concept could be expanded to include *Poaceae* crops in the future, thereby increasing its universality and application potential.

Outstanding questions box

- The *Poaceae* plants are an ideal group for extending this model, and additional research on their root development, exudates, and functional microbiome is imperative to update the model.
- How should the host exudates be altered to more efficiently modulate functional microbiome that increase plant growth and health? In addition, is there a circadian rhythm in aerial root-mucilage exudates and microbiome?
- Can natural plant functional metabolites (exudates) be utilized more effectively, such as in the form of seed coatings?
- Can aerial root mucilage diazotrophs be utilized to augment the nitrogen uptake efficiency of cereal crops?

## Concluding remarks and perspectives

Root exudates are important interfaces for host–microbiome interactions. However, the functional interactions mediated by root exudates are not fully understood. The aerial roots of many wild plants and cereal crops, such as maize and sorghum, can secrete large amounts of mucilage which is a specific type of root exudates, but has been a neglected biological phenomenon. Previous studies have shown that mucilage, which is rich in carbohydrates and potential antibiotics, provides an ideal niche for diazotrophs, and defense against pathogen invasion. Moreover, there also seem to be gatekeeper microbes in mucilage that can help the host resist the invasion of pathogenic and environmental microbes. However, how these diazotrophic and gatekeeper microbes are recruited by the host and how they function to maintain the homeostasis of the microenvironment remain unclear. To answer these scientific questions, we propose a research model based on aerial root mucilage probiotics, which are typical root exudate–functional microbiome. To identify special metabolites that recruit or shape the special and functional microbes in the special niche of the aerial root mucilage microenvironment. Herein, we propose the aerial root mucilage probiotics system as a novel model for identifying the crucial interactions between functional metabolites and the probiotic microbiome and for determining how probiotics, including diazotrophic and functional microbiomes, are selected for use in this system.

Overall, this special model provides new insight into how plants engage with probiotics while restricting pathogens through root exudation. Similarly, the new biological functions of aerial roots and the traditional definition of the rhizosphere should be further considered. We hope that the aerial root mucilage–functional microbiome study system will provide basic biological insights into biological interactions.

### Glossary

Rhizosphere: The environmental zone (~1–3 mm) around roots, which includes exudates and microbiota. In this study, the rhizosphere includes the underground or aerial root system in the surrounding soil and aerial environment.

Homeostasis: The original definition of homeostasis is a self-regulatory process in which a biological system tends to remain stable while adjusting to optimal survival conditions. Herein, homeostasis here refers to the combination of host factors and functional microbiota that shape the dominant diazotrophs in the mucilage system rather than the massive proliferation and “large burst” of pathogenic and other environmental microbes. The stable interactions between host plants, the functional microbiome and environmental microbes: this definition also implies that there are stable interactions between host plants, the functional microbiota, and environmental microbes.

Functional microbiome: Microbes that promote host plant growth and defense against pathogens and can be equated with functional probiotics. These microbes can be defined as a group that consists of diazotrophs and pathogen defense (biocontrol or gatekeeper microbes) bacteria and fungi in the aerial root mucilage probiotic system.

Functional metabolites: Key root exudate compounds that “recruit” or enrich functional microbiota (prebiotics) and/or defend against pathogenic and environmental microbes (antibiotics) and can regulate the rhizosphere microbial community and homeostasis.

Gatekeeper/police microbes: Microbes that inhibit unwanted pathogenic and environmental microbes and are friendly to the host and the sympatric functional microbiota. These strains can be used as biocontrol strains in agricultural applications.

*Microbiome* gene (*M* gene): Gene targets for host regulation of plant-associated microbial communities. Plant microbiome-modulating genes may be distributed and conserved in multi-hosts, hence they are referred to as “*M*-gene”.

## Supplementary Material

Supplementary_Figure_1-2_wrae223

Supplementary_Table_1-2_wrae223

## Data Availability

All the data generated or analyzed during this study are included in this published article.

## References

[1] Venturi V , Keel C. Signaling in the rhizosphere. *Trends Plant Sci* 2016;21:187–98. 10.1016/j.tplants.2016.01.005 26832945

[2] Pang Z , Chen J, Wang T et al. Linking plant secondary metabolites and plant microbiomes: a review. *Front Plant Sci* 2021;12:621276. 10.3389/fpls.2021.621276 33737943 PMC7961088

[3] Chai YN , Schachtman DP. Root exudates impact plant performance under abiotic stress. *Trends Plant Sci* 2022;27:80–91. 10.1016/j.tplants.2021.08.003 34481715

[4] Ehlers BK , Berg MP, Staudt M et al. Plant secondary compounds in soil and their role in belowground species interactions. *Trends Ecol Evol* 2020;35:716–30. 10.1016/j.tree.2020.04.001 32414604

[5] Bennett AB , Pankievicz VCS, Ane JM. A model for nitrogen fixation in cereal crops. *Trends Plant Sci* 2020;25:226–35. 10.1016/j.tplants.2019.12.004 31954615

[6] Sasse J , Martinoia E, Northen T. Feed your friends: do Plant exudates shape the root microbiome? *Trends Plant Sci* 2018;23:25–41. 10.1016/j.tplants.2017.09.003 29050989

[7] Yu P , He X, Baer M et al. Plant flavones enrich rhizosphere Oxalobacteraceae to improve maize performance under nitrogen deprivation. *Nat Plants* 2021;7:481–99. 10.1038/s41477-021-00897-y 33833418

[8] Zhou X , Zhang J, Khashi u Rahman M et al. Interspecific plant interaction via root exudates structures the disease suppressiveness of rhizosphere microbiomes. *Mol Plant* 2023;16:849–64. 10.1016/j.molp.2023.03.009 36935607

[9] Koprivova A , Schuck S, Jacoby RP et al. Root-specific camalexin biosynthesis controls the plant growth-promoting effects of multiple bacterial strains. *Proc Natl Acad Sci USA* 2019;116:15735–44. 10.1073/pnas.1818604116 31311863 PMC6681745

[10] Stringlis IA , Yu K, Feussner K et al. MYB72-dependent coumarin exudation shapes root microbiome assembly to promote plant health. *Proc Natl Acad Sci USA* 2018;115:E5213–22. 10.1073/pnas.1722335115 29686086 PMC5984513

[11] Paasch BC , He SY. Toward understanding microbiota homeostasis in the plant kingdom. *PLoS Pathog* 2021;17:e1009472. 10.1371/journal.ppat.1009472 33886694 PMC8061798

[12] Sma-Air S , Ritchie RJ. Photosynthesis in a Vanda sp orchid with photosynthetic roots. *J Plant Physiol* 2020;251:153187. 10.1016/j.jplph.2020.153187 32505060

[13] Zotz G , Winkler U. Aerial roots of epiphytic orchids: the velamen radicum and its role in water and nutrient uptake. *Oecologia* 2013;171:733–41. 10.1007/s00442-012-2575-6 23292456

[14] Nazari M . Plant mucilage components and their functions in the rhizosphere. *Rhizosphere* 2021;18:100344. 10.1016/j.rhisph.2021.100344

[15] Van Deynze A , Zamora P, Delaux PM et al. Nitrogen fixation in a landrace of maize is supported by a mucilage-associated diazotrophic microbiota. *PLoS Biol* 2018;16:e2006352. 10.1371/journal.pbio.2006352 30086128 PMC6080747

[16] Amicucci MJ , Galermo AG, Guerrero A et al. Strategy for structural elucidation of polysaccharides: elucidation of a maize mucilage that harbors diazotrophic bacteria. *Anal Chem* 2019;91:7254–65. 10.1021/acs.analchem.9b00789 30983332

[17] Xu S , Li XQ, Guo H et al. Mucilage secretion by aerial roots in sorghum (*Sorghum bicolor*): sugar profile, genetic diversity, GWAS and transcriptomic analysis. *Plant Mol Biol* 2023;112:309–23. 10.1007/s11103-023-01365-1 37378835

[18] Pang Z , Mao X, Zhou S et al. Microbiota-mediated nitrogen fixation and microhabitat homeostasis in aerial root-mucilage. *Microbiome* 2023;11:85. 10.1186/s40168-023-01525-x 37085934 PMC10120241

[19] Guo K , Yang J, Yu N et al. Biological nitrogen fixation in cereal crops: progress, strategies, and perspectives. *Plant Commun* 2023;4:100499. 10.1016/j.xplc.2022.100499 36447432 PMC10030364

[20] Nazari M , Bickel S, Benard P et al. Biogels in soils: plant mucilage as a biofilm matrix that shapes the rhizosphere microbial habitat. *Front Plant Sci* 2021;12:798992. 10.3389/fpls.2021.798992 35095970 PMC8792611

[21] Rafael EV , Jennifer W, Vania P et al. Mucilage produced by sorghum aerial roots supports a nitrogen-fixing community. *bioRxiv* 2023 2023.08.05.552127. 10.1101/2023.08.05.552127

[22] Higdon SM , Pozzo T, Kong N et al. Genomic characterization of a diazotrophic microbiota associated with maize aerial root mucilage. *PLoS One* 2020;15:e0239677–7. 10.1371/journal.pone.0239677 32986754 PMC7521700

[23] Higdon SM , Pozzo T, Tibbett EJ et al. Diazotrophic bacteria from maize exhibit multifaceted plant growth promotion traits in multiple hosts. *PLoS One* 2020;15:e0239081. 10.1371/journal.pone.0239081 32925972 PMC7489573

[24] Pozzo T , Higdon SM, Pattathil S et al. Characterization of novel glycosyl hydrolases discovered by cell wall glycan directed monoclonal antibody screening and metagenome analysis of maize aerial root mucilage. *PLoS One* 2018;13:e0204525. 10.1371/journal.pone.0204525 30256843 PMC6157868

[25] Bloch SE , Ryu MH, Ozaydin B et al. Harnessing atmospheric nitrogen for cereal crop production. *Curr Opin Biotech* 2020;62:181–8. 10.1016/j.copbio.2019.09.024 31790876

[26] Gao J , Feng P, Zhang J et al. Enhancing maize’s nitrogen-fixing potential through ZmSBT3, a gene suppressing mucilage secretion. *J Integr Plant Biol* 2023;65:2645–59. 10.1111/jipb.13581 37929676

[27] Mechan-Llontop ME , Mullet J, Shade A. Phyllosphere exudates select for distinct microbiome members in sorghum epicuticular wax and aerial root mucilage. *Phytobiomes J* 2023;7:184–97. 10.1094/pbiomes-08-22-0046-fi

[28] Mechan-Llontop ME , Mullet J, Shade A. Genome-sequenced bacterial collection from sorghum epicuticular wax. *Microbiol Resour Announc* 2023;12:e0048423. 10.1128/MRA.00484-23 37909721 PMC10720456

[29] Chepsergon J , Moleleki LN. Rhizosphere bacterial interactions and impact on plant health. *Curr Opin Microbiol* 2023;73:102297. 10.1016/j.mib.2023.102297 37002974

[30] Paasch BC , Sohrabi R, Kremer JM et al. A critical role of a eubiotic microbiota in gating proper immunocompetence in Arabidopsis. *Nat Plants* 2023;9:1468–80. 10.1038/s41477-023-01501-1 37591928 PMC10505558

[31] Song S , Morales Moreira Z, Briggs AL et al. PSKR1 balances the plant growth–defence trade-off in the rhizosphere microbiome. *Nat Plants* 2023;9:2071–84. 10.1038/s41477-023-01539-1 37973937

[32] Song Y , Wilson AJ, Zhang X-C et al. FERONIA restricts Pseudomonas in the rhizosphere microbiome via regulation of reactive oxygen species. *Nat Plants* 2021;7:644–54. 10.1038/s41477-021-00914-0 33972713

[33] Wolinska KW , Vannier N, Thiergart T et al. Tryptophan metabolism and bacterial commensals prevent fungal dysbiosis in *Arabidopsis* roots. *Proc Natl Acad Sci USA* 2021;118:e2111521118. 10.1073/pnas.2111521118 34853170 PMC8670527

[34] Ma KW , Niu Y, Jia Y et al. Coordination of microbe-host homeostasis by crosstalk with plant innate immunity. *Nat Plants* 2021;7:814–25. 10.1038/s41477-021-00920-2 34031541 PMC8208891

[35] He D , Singh SK, Peng L et al. Flavonoid-attracted Aeromonas sp. from the Arabidopsis root microbiome enhances plant dehydration resistance. *ISME J* 2022;16:2622–32. 10.1038/s41396-022-01288-7 35842464 PMC9561528

[36] Tian B , Pei Y, Huang W et al. Increasing flavonoid concentrations in root exudates enhance associations between arbuscular mycorrhizal fungi and an invasive plant. *ISME J* 2021;15:1919–30. 10.1038/s41396-021-00894-1 33568790 PMC8245413

[37] Korenblum E , Massalha H, Aharoni A. Plant-microbe interactions in the rhizosphere via a circular metabolic economy. *Plant Cell* 2022;34:3168–82. 10.1093/plcell/koac163 35678568 PMC9421461

[38] Yan D , Tajima H, Cline LC et al. Genetic modification of flavone biosynthesis in rice enhances biofilm formation of soil diazotrophic bacteria and biological nitrogen fixation. *Plant Biotechnol J* 2022;20:2135–48. 10.1111/pbi.13894 35869808 PMC9616522

[39] Chakraborty S , Venkataraman M, Infante V et al. Scripting a new dialogue between diazotrophs and crops. *Trends Microbiol* 2024;32:577–89. 10.1016/j.tim.2023.08.007 37770375 PMC10950843

[40] Voges M , Bai Y, Schulze-Lefert P et al. Plant-derived coumarins shape the composition of an Arabidopsis synthetic root microbiome. *Proc Natl Acad Sci USA* 2019;116:12558–65. 10.1073/pnas.1820691116 31152139 PMC6589675

[41] Getzke F , Thiergart T, Hacquard S. Contribution of bacterial-fungal balance to plant and animal health. *Curr Opin Microbiol* 2019;49:66–72. 10.1016/j.mib.2019.10.009 31731228

[42] Weston LA , Mathesius U. Flavonoids: their structure, biosynthesis and role in the rhizosphere. *Including Allelopathy J Chem Ecol* 2013;39:283–97. 10.1007/s10886-013-0248-5 23397456

[43] Arnault G , Mony C, Vandenkoornhuyse P. Plant microbiota dysbiosis and the Anna Karenina principle. *Trends Plant Sci* 2023;28:18–30. 10.1016/j.tplants.2022.08.012 36127241

[44] Spragge F , Bakkeren E, Jahn MT et al. Microbiome diversity protects against pathogens by nutrient blocking. *Science* 2023;382:eadj3502. 10.1126/science.adj3502 38096285 PMC7616675

[45] Kost C , Patil KR, Friedman J et al. Metabolic exchanges are ubiquitous in natural microbial communities. *Nat Microbiol* 2023;8:2244–52. 10.1038/s41564-023-01511-x 37996708

[46] Ruan C , Ramoneda J, Gogia G et al. Fungal hyphae regulate bacterial diversity and plasmid-mediated functional novelty during range expansion. *Curr Biol* 2022;32:5285–5294.e4. 10.1016/j.cub.2022.11.009 36455559

[47] Wilde J , Slack E, Foster KR. Host control of the microbiome: mechanisms, evolution, and disease. *Science* 2024;385:eadi3338. 10.1126/science.adi3338 39024451

[48] Delaux PM , Schornack S. Plant evolution driven by interactions with symbiotic and pathogenic microbes. *Science* 2021;371:eaba6605. 10.1126/science.aba6605 33602828

[49] Zhao Y , Zhang R, Jiang KW et al. Nuclear phylotranscriptomics and phylogenomics support numerous polyploidization events and hypotheses for the evolution of rhizobial nitrogen-fixing symbiosis in Fabaceae. *Mol Plant* 2021;14:748–73. 10.1016/j.molp.2021.02.006 33631421

[50] Escudero-Martinez C , Bulgarelli D. Tracing the evolutionary routes of plant–microbiota interactions. *Curr Opin Microbiol* 2019;49:34–40. 10.1016/j.mib.2019.09.013 31698159

[51] Levy A , Salas Gonzalez I, Mittelviefhaus M et al. Genomic features of bacterial adaptation to plants. *Nat Genet* 2018;50:138–50. 10.1038/s41588-017-0012-9 PMC5957079 29255260

[52] Li M-H , Liu K-W, Li Z et al. Genomes of leafy and leafless Platanthera orchids illuminate the evolution of mycoheterotrophy. *Nat Plants* 2022;8:373–88. 10.1038/s41477-022-01127-9 35449401 PMC9023349

[53] Ben-Tov D , Idan-Molakandov A, Hugger A et al. The role of COBRA-LIKE 2 function, as part of the complex network of interacting pathways regulating Arabidopsis seed mucilage polysaccharide matrix organization. *Plant J* 2018;94:497–512. 10.1111/tpj.13871 29446495

[54] Atwell S , Huang YS, Vilhjalmsson BJ et al. Genome-wide association study of 107 phenotypes in Arabidopsis thaliana inbred lines. *Nature* 2010;465:627–31. 10.1038/nature08800 20336072 PMC3023908

[55] Deng S , Caddell DF, Xu G et al. Genome wide association study reveals plant loci controlling heritability of the rhizosphere microbiome. *ISME J* 2021;15:3181–94. 10.1038/s41396-021-00993-z 33980999 PMC8528814

[56] Escudero-Martinez C , Coulter M, Alegria Terrazas R et al. Identifying plant genes shaping microbiota composition in the barley rhizosphere. *Nat Commun* 2022;13:3443. 10.1038/s41467-022-31022-y 35710760 PMC9203816

[57] Brachi B , Filiault D, Whitehurst H et al. Plant genetic effects on microbial hubs impact host fitness in repeated field trials. *Proc Natl Acad Sci USA* 2022;119:e2201285119. 10.1073/pnas.2201285119 35867817 PMC9335298

