# Supplementary material for: Probiotic model for studying rhizosphere interactions of root exudates and the functional microbiome
Source: ISME J. 2024 Nov 4;18(1):wrae223. doi: 10.1093/ismejo/wrae223 (PMC11572495; doi:10.1093/ismejo/wrae223)
Supplement: Supplementary_Figure_1-2_wrae223 [file supplementary_figure_1-2_wrae223.docx]

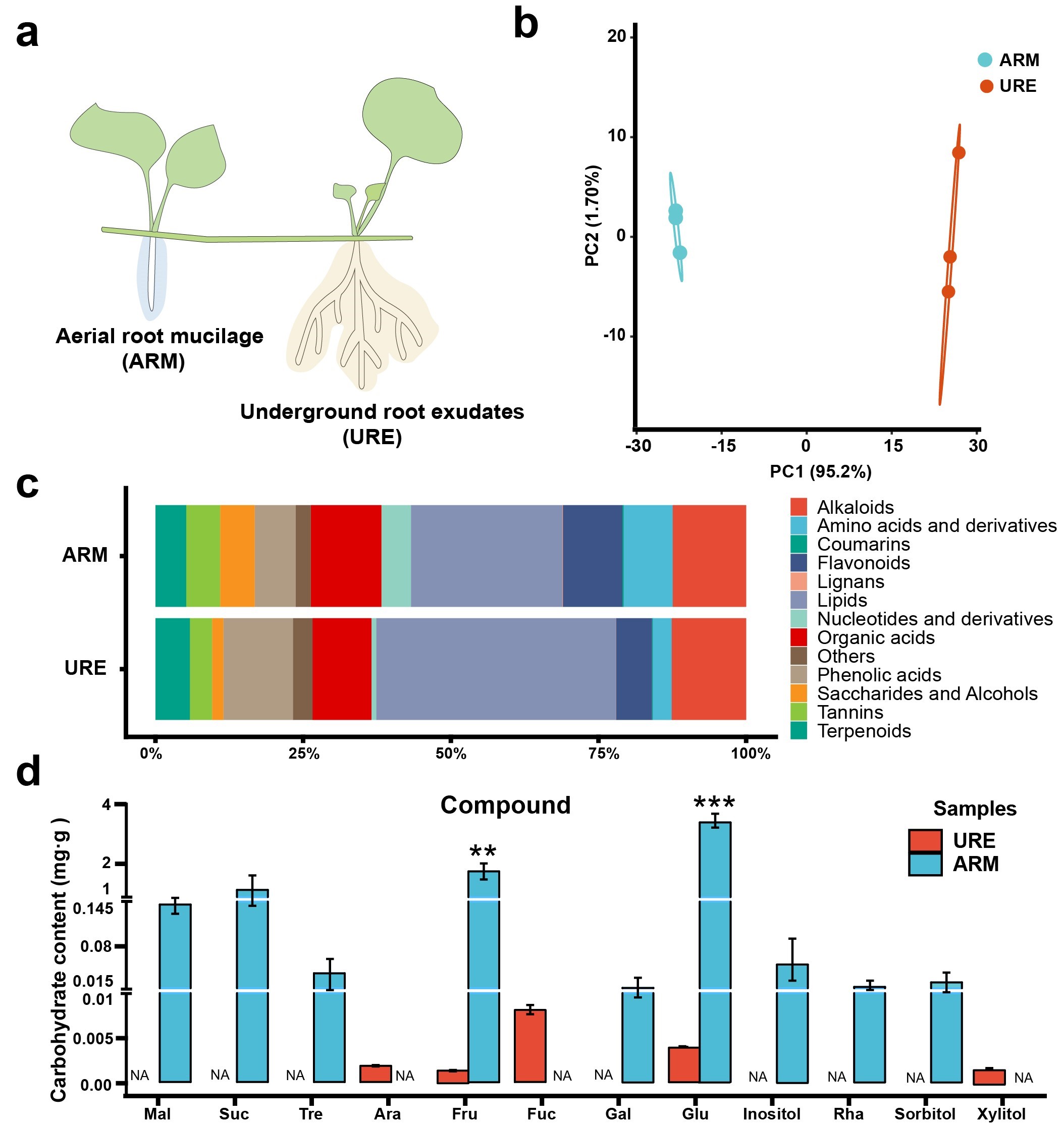


**Fig. S1 Metabolites and carbohydrate compounds of aerial root mucilage and underground root exudates of dicotyledon (*Melastomataceae*) plants (*Heterotis rotundifolia*) (Pang et al., 2023).**





**Fig. S2 Bacterial isolates from four aerial root mucilage plants*.***
